# A Novel Nitrogen Enriched Hydrochar Adsorbents Derived from Salix Biomass for Cr (VI) Adsorption

**DOI:** 10.1038/s41598-018-21238-8

**Published:** 2018-03-06

**Authors:** Yanqiu Lei, Haiquan Su, Fuli Tian

**Affiliations:** School of Chemistry & Chemical Engineering, Inner Mongolia University, 235 West College Road, Hohhot, 010021 Inner Mongolia, China

## Abstract

Hydrochars were prepared from Salix by hydrothermal carbonization, and characterized by FT-IR, ^13^C NMR, XPS, UV-vis, TG, SEM and BET techniques. The results showed that the hydrochars with molecular sieve-type open pore structure contained numbers of oxygen and nitrogen functional groups, which benefited the adsorption and diffusion of adsorbent Cr (VI). The hydrochar obtained from 26 h reaction (HC-26) was indicated an excellent adsorbent compared to the commercial activated carbon, and its maximum removal efficiency for Cr (VI) reaches up to 99.84% at pH 1. Langmuir´s model is well fitted the experimental equilibrium adsorption data of total Cr. The bath experiment results showed that Cr (VI) could be removed rapidly in the first 300 min. Furthermore, the adsorption kinetics process of HC-26 could be described by pseudo-second-order model. Based on the above results, HC-26 could be acted as a potential efficient adsorbent for removal of Cr (VI) from aqueous solution.

## Introduction

The swift industrialization has led to enormous economic growth as well as increasing awareness of the ecological effects of toxic metals and other bio-magnification on food. More and more attentions have been arisen in environmental science due to the widespread pollution of heavy metals, i.e. their toxicity, non-degradability, accumulation in living organisms, and persistent threat to human lives^1–3^. Chromium is one of the most highly toxic pollutants among these heavy metals, which have been generated from industrial wastewater from electroplating, leather tanning, textiles, printing, dying, and etc^4,5^. On the other hand, Cr (VI) exists in the form of soluble and mobile chromate ions (HCrO_4_^−^ or Cr_2_O_7_^2−^), therefore, they can transfer freely in aqueous environment. It is well known that certain types of cancer and health problems have been caused by persistent exposure to Cr (VI) in the digestive tract, lungs and other organs^6,7^. Therefore, it is crucial to remove Cr (VI) from contaminated aqueous solution before disposal^8–14^ in order to maintain the health living environment. So, it is great challenging for us to develop both economical and eco-friendly methods for Cr (VI) treatment.

Adsorption is a simple, high efficiency, economical and convenient method for removing trace metals from aqueous solution. A variety of materials has been used as Cr (VI) sorbents, including activated carbons, biological materials, polymer resins, zeolites, mineral, and industrial wastes^15–19^. Among the different adsorbents, activated carbon has been studied extensively, due to its large surface area and high porosity. However, the adsorption effectiveness of adsorbents is not only dependent on specific surface area, pore structure, but also the presence of surface functional groups. A large number of studies have shown that the adsorption of Cr (VI) by activated carbon is closely related to the interaction between metal ions and its surface functional groups, for example, nitrogen containing functional groups not only chelates cationic metal ions, but also adsorbs anionic metal species through electrostatic interaction or hydrogen bonding^20–22^. Thus, many research works have been done to modify the surface properties of activated carbon by various methods, such as oxidation, sulfuration, ammonification, and coordinated ligand anchorage, even impregnating other metal oxide to improve the affinity of chromium toward adsorbents^23–25^. However, the modification procedures of activated carbon are complex and even unconventional precursors has been used to carbonization in order to reach optimum result^26^. Consequently, the conspicuous problem concerning modification of activated carbon lies within its large densities, or inferior adsorption. So, it is necessary to find a cost-effective and simple way to prepare adsorbent with a large number of surface functional groups from natural sources or wastes from industrial and agricultural processes. Therefore, both the environment and commercial value could be benefited from the new type of the adsorbent.

Recently, considerable researches have focused on synthesis of biomass-based carbon materials by hydrothermal carbonization (HTC)^27,28^. HTC is a promising, inexpensive, low energy consumption approach for converting biomass, or its derivatives into new type of carbon-based materials (hydrochars) at relatively mild conditions^29^. In HTC process, because water is used as the reaction medium, and “wet” biomass may be utilized without dried before the HTC process. Therefore, HTC is considered as a simple and efficient process, and its cost, chemical usage and pollution could be significantly reduced compared to the other traditional techniques. What’s more, hydrochar is typically rich in oxygen-containing functional groups on its surface, such as hydroxyl, carbonyl and carboxyl groups, and these groups which constitute the majority of the adsorption sites have a high affinity for metal ions^30^. Previous studies suggested hydrochars can be used as adsorbents to remove heavy metals in industrial effluents, owing to number of oxygen and nitrogen functionalities on their surface^31–34^. Wherein, a certain amount of nitrogen containing surface functional group on hydrochar would endow it the better adsorption performance than that of ordinary one.

The objective of this work was to present the hydrothermal syntheses of hydrochars with surface oxygen and nitrogen functional groups from biomass-Salix. Salix is short-rotation coppice and used as sand-fixing in Gobi desert^35,36^. Salix is rich in phenolic constituents, a lot of oxygen and nitrogen functionalities may be introduced into the hydrochar network during HTC, which is considered as an *in situ* “one-step” functionalization process. The hydrochars by HTC showed a stable molecular sieve-type open pore structure without any further treatments, and this unique structure in hydrochar is generated from the special texture of Salix^36^.

## Results and Discussion

### Characterization of Hydrochar

#### FT-IR and NMR Analysis

**Figure 1:**
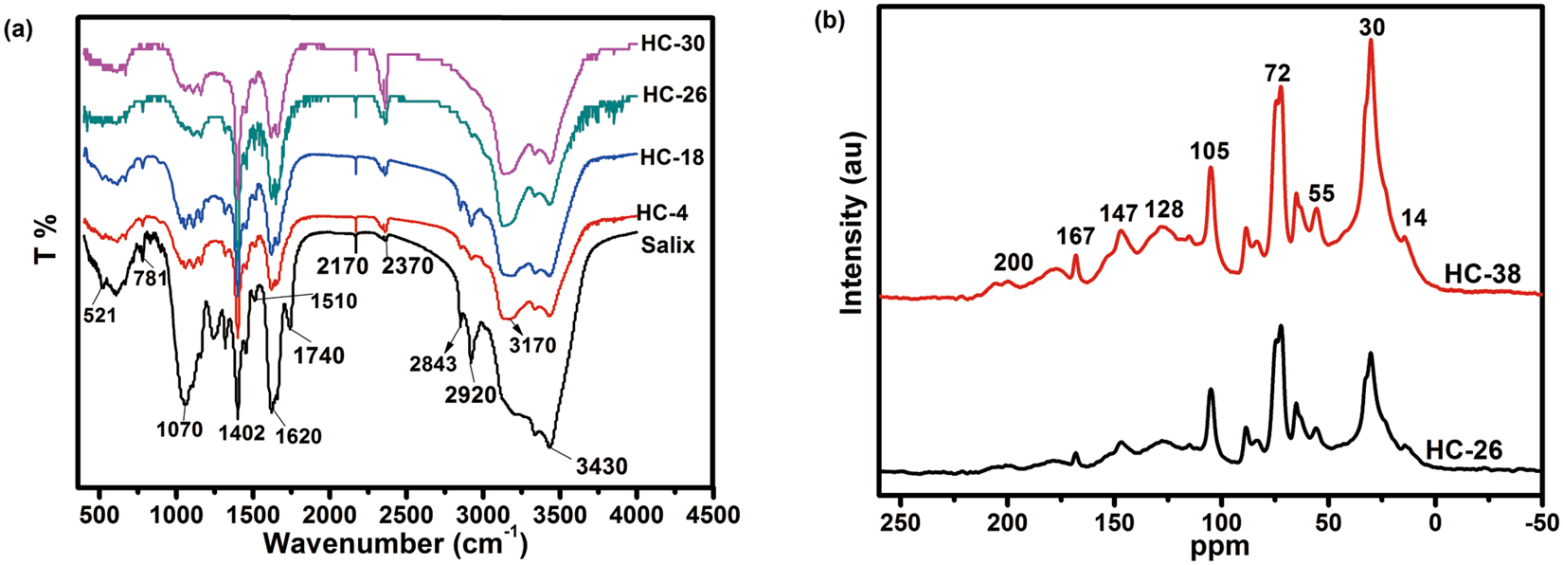
FT-IR spectra of the Salix and hydrochars obtained at different time (**a**) and ^13^C CP-MAS NMR spectra of hydrochars for 26 h and 38 h (**b**).

The transformations of chemical functional groups in hydrochars were characterized by Fourier-transform infrared (FT-IR) and NMR spectroscopy. As shown in Fig. 1a, the IR spectra of hydrochars were changed with reaction time. In the range of 3430–3170 cm^−1^, overlapping peak bands of O-H and N-H stretching vibrations were observed for all hydrochar samples. The O-H adsorption peaks become weaker and narrower with increase of reaction time, which might be attributed to the dehydration of hydrochar, meanwhile, intensities of N-H bands were increased. Absorbance peaks between 2920 cm^−1^ and 2843 cm^−1^ represent saturated aliphatic C-H stretching vibrations. The peaks disappeared as the reaction time increased to 30 h, which showed the the alkyl chain has been decomposed. The bands at 2170–2370 cm^−1^ are assigned to the C≡N stretching vibrations, and 1400 cm^−1^ belong to the characteristic adsorptions of C-N, and its intensities increased with the reaction time extended. The reason is that nitrogen is still fixed in the hydrochar network in the form of nitrogen compounds, but carbohydrates have been hydrolyzed in biomass^37^. The bands at 1740 cm^−1^ and 1620 cm^−1^ correspond to C=O and C=C groups vibrations respectively. From the Fig. 1a, we can tell the peak of 1740 cm^−1^ almost disappeared after the reaction time extended to more than 4 h, which showed that the de-carbonyl reaction could be gradually taken place as HTC process was extended. The bands around the 1070 cm^−1^ region mainly correspond to C-O-C stretching and O-H bending vibrations. The peak intensity for the peak of C-O-C and O-H was reduced with increase of reaction time, which indicated that dehydration and aromatization processes happened during HTC.

The high-field ^13^C CP-MAS NMR spectra of hydrochars were shown in Fig. 1b. The spectra of HC-26 and HC-38 were similar, nevertheless, the relative intensities of peaks exhibited some differences. The weak signals at 14, 55 and 147 ppm were attributed to adsorption of the residual lignin^38,39^, while the peak at 75 ppm could be ascribed to adsorption of cellulose. The presence of these peaks indicated that there were still residual part of lignin and cellulose in hydrochars after HTC. The peak at around 30 ppm corresponded to the aliphatic carbon atoms, wherein, the peak at 128 ppm could be unambiguously ascribed to the protonated carbon atoms of aromatic rings^40^. These signals at 30 ppm and 128 ppm in HC-38 were more intense than that in HC-26, indicating that an amount of aromatic structure has been gradually formed with extension of reaction time. The peaks at 105, 114 and 167 ppm were assigned to furanic groups^41^. From these spectrum, we can easily tell that the formation of aromatic structure of hydrochar was much affected by the reaction time.

### XPS Analysis

To further insight into the surface chemical compositions of the hydrochars, X-ray photo electron spectroscopy (XPS) was recorded. Figure S1 showed the C 1 s envelopes of hydrochars for 26 h and 38 h as representative examples. The C 1 s spectrum contains four signals, corresponding to C=C/C-Hx/C-C (284.6 ± 0.2 eV, C1), C–O (286.3 ± 0.3 eV, C2), C=O (287.7 ± 0.2 eV, C3) and O-C=O (288.1 ± 0.1 eV, C4)^42^. Compared with that of hydrochar for HC-26, all the peak intensities of C-O, C=O and O-C=O groups were decreased in the sample of HC-38, which indicated that substantial amount of oxygen-containing functional groups took part in the reaction. The percentages of C, N and O in the surface of hydrochars calculated by XPS were listed Table 1. It can be seen that the percentages of C and N increased as the reaction time increase, at the same time, there was a reduction in the percentages of O. These changes suggested the dehydration and/or decarboxylation reaction took place during hydrothermal carbonization process. It is worth mentioning that HC-26 contain more surface functional groups including oxygen and nitrogen-containing functional groups than that of others.

**Table 1:** Textural properties and chemical elemental analyses of hydrochars.

Sample	C1s	N1s	O1s	S_BET_ (m^2^ g^−1^)	V_t_ (m^3^ g^−1^)	Da (nm)
Salix	81.6	1.2	17.2	—	—	—
HC-6	70.4	1.0	28.6	6.9	0.025	197.6
HC-16	71.0	1.1	27.9	13.1	0.045	175.8
HC-26	74.3	1.5	24.2	15.1	0.067	260.0
HC-34	75.9	1.5	22.6	14.6	0.066	243.6
HC-38	79.1	1.8	19.1	18.3	0.074	213.9

S_BET_: BET surface area; V_t_: Total pore volume calculated as the amount of nitrogen adsorbed at the relative pressure of 0.97; Da: Average pore diameter; C1s, N1s, O1s: The content of C, N, and O calculated by XPS.

### TG Analysis

**Figure 2:**
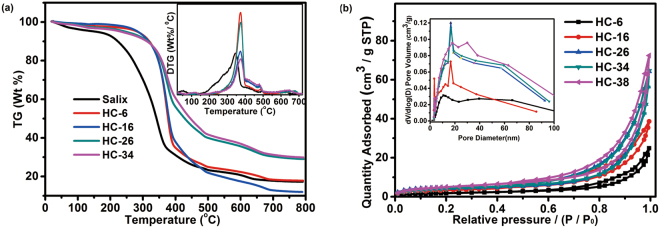
TG and DTG curves (**a**) and Nitrogen adsorption-desorption isotherms and corresponding pore size distributions (**b**) of hydrochars obtained at different time.

TG and DTG analyses of Salix and the hydrochars were displayed in Fig. 2a. Some differences were observed on all the samples from the TG curves. The initial decomposition temperatures of hydrochars are higher than that of Salix, which reflected differences in the inherent structural properties and composition in the both types of samples. Salix is consisted of three major components which are hemicellulose, cellulose, and lignin. The decomposition of main components hemicellulose and cellulose occurred in the range of 200 °C −380 °C, thus, weight loss of Salix has been observed clearly. When the decomposition temperature was higher than 380 °C, the weight loss is much slower, which was attributed to the decomposition of lignin. Salix’s major components changed with the reaction time of HTC. However, the thermal stabilities of HC-6 and HC-16 were obviously higher than that of Salix, but were lower than that of HC-26 and HC-34. The reason was that the hemicellulose and amorphous part of cellulose could be hydrolyzed to oligomer/monosaccharide, thereafter, resulting in increasing the crystallinity of cellulose so that the thermal stability of hydrochar has been increased as well. However, because of the shorter reaction time, the hydrolysis products of hemicellulose and cellulose had not got enough time to be polymerized/condensed, a lot of oligomers/monosaccharides were involved in hydrochar, which made the thermal stability of HC-6 and HC-16 lower than that of the HC-26 and HC-34. It was found from the TG curves that a significant decay of weight happened around 286 °C for HC-26 and HC-34, and their decompositions were taken place in a low mass loss rate, which revealed hydrochars’ structure has been much influenced by HTC reaction time. The distributions of DTG for the samples are also investigated in Fig. 2a appended. The main unsymmetrical peaks of Salix appeared at around 345 °C, corresponding to decomposition of cellulose and hemicellulose in Salix^43^. After HTC, the temperature of cellulose decomposition in hydrochars shifted to higher temperature than that of Salix, which might be attributed to the increase of crystallinity in cellulose. Meanwhile, From the peaks of HC-6 and HC-16 in Fig. 2a appended, it is observed that mass loss rate is higher than that of Salix. Several small peaks were observed beyond 345 °C, and these peaks indicated that decomposition of lignin was happened, however, the decomposition temperature of lignin in hydrochar was lower than that of Salix, implying that the original framework structure of lignin was disrupted, and its stability was decreased.

**Figure 3:**
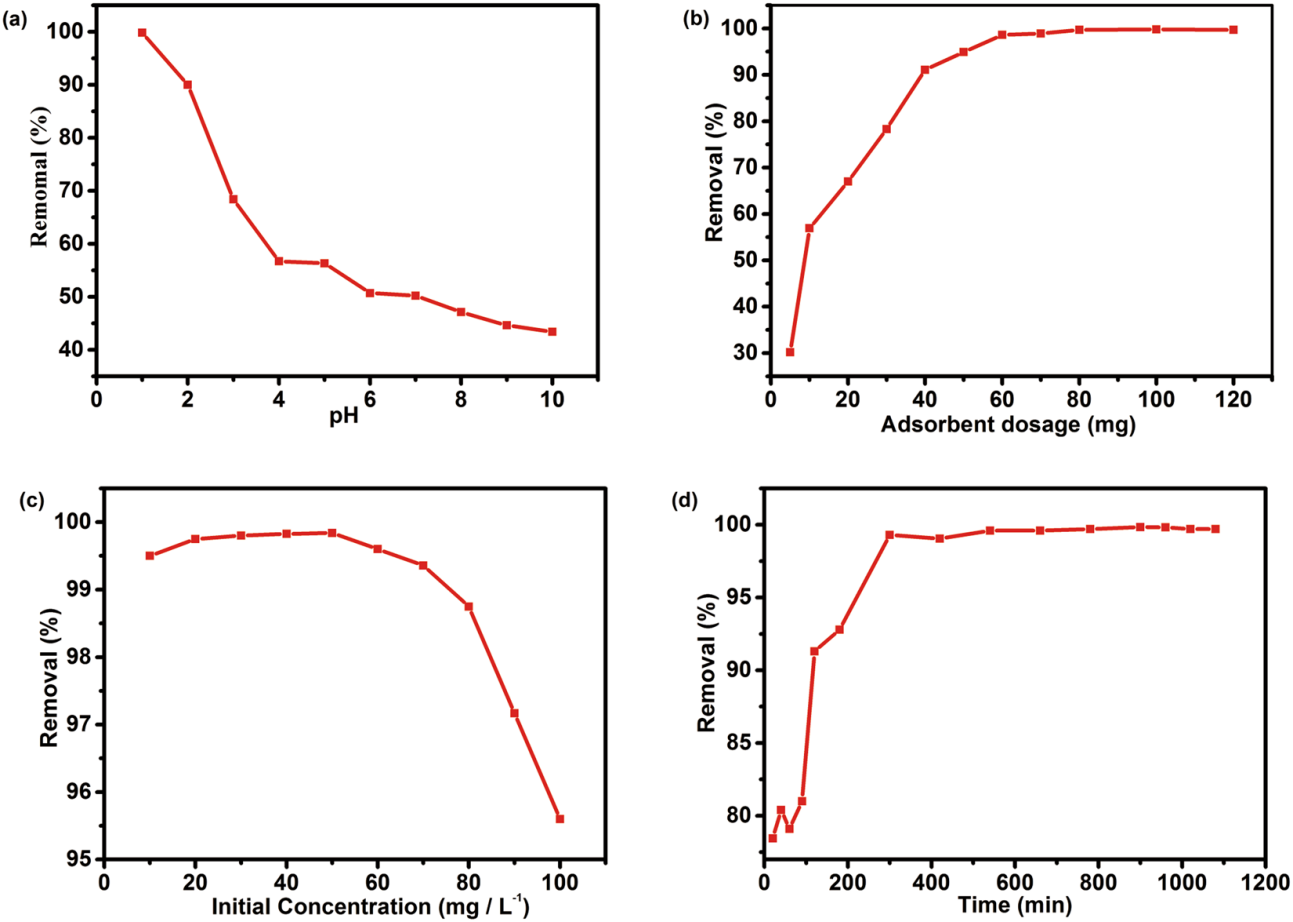
Effects of initial pH (**a**), adsorbent dosage(**b**), initial concentration(**c**) and contact time (**d**) on adsorption of Cr(VI) by hydrochar HC-26 (under the conditions: rotate speed 180 rpm, temperature:20 °C, contact time 15 h).

### BET Analysis

The textural properties of the hydrochars were analyzed by nitrogen sorption. The N_2_ adsorption/desorption isotherms and corresponding pore size distributions for the hydrochars were shown in Fig. 2b. All isotherms display Type I/IV isotherms with hysteresis loops, indicating that all hydrochars mainly possess mesoporous and macroporous structure character. The molecular sieve-type pore structure was formed after 26 h, and the mesopore structure has been shifted to that of larger pore (shown in Fig. 2b of 26–38 h), which was further verified by the pore size distributions of all hydrochars in Fig. 2b appended. The textural properties of the hydrochars were listed in Table 1. From which the specific BET surface area and total pore volume showed a trend of increase with the extension of the reaction time, however, they did not change significantly when the reaction time was extended to more than 26 h. The reason is that the liquid phase products formed on the initial HTC stage blocked hydrochars’ pores, which indicated their specific surface area has been reduced, and then the specific surface area increased gradually with extension of the reaction time, attributed to the formation of hydrochar which is caused by repolymerization of the components in liquid phase.

**Figure 4:**
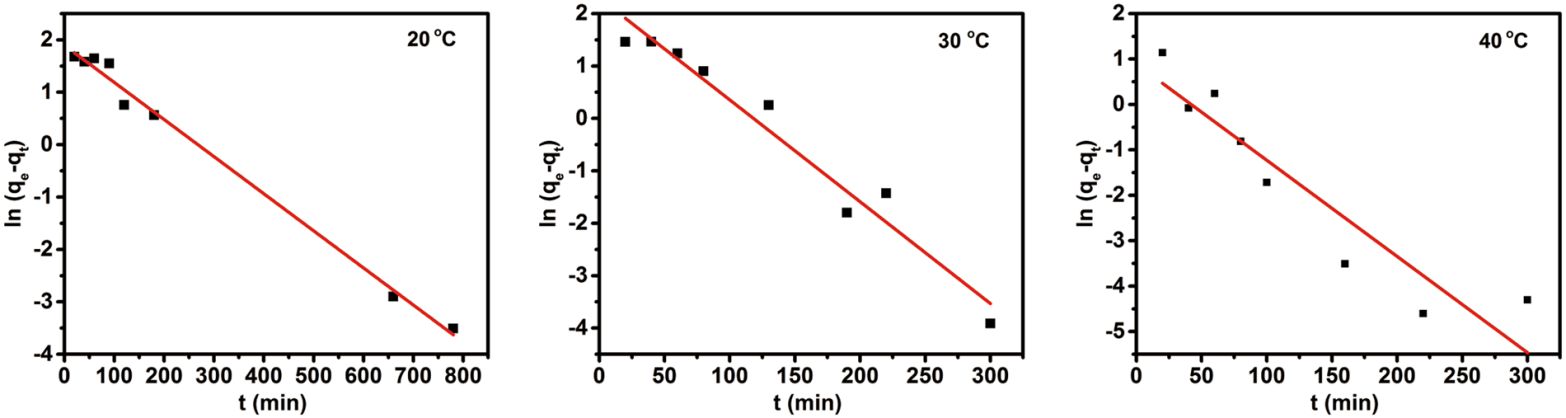
The kinetic fitting plots by pseudo-first-order equation at different temperature.

**Figure 5:**
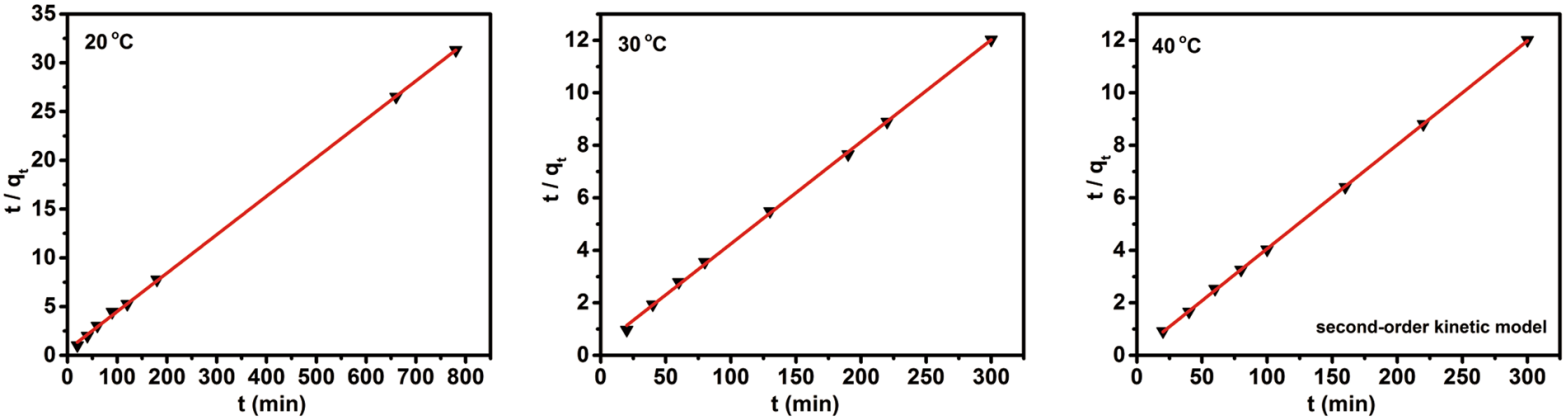
The kinetic fitting plots by pseudo-second-order equation at different temperature.

### SEM Analysis

SEM results have been used to evaluate the morphology variations of hydrochars with reaction time, as showed in Figure S2. It could be clearly seen that a large amount of small cavities structures were formed on fiber surface which is attributed to the dissolution of hemicellulose after reaction of 6 h. With the extension of reaction time, the amorphous cellulose and some soluble segments of lignin were gradually hydrolyzed, and then the hydrolysis products were subsequently polymerized/recondensed, which large number of sphere-like morphology and irregular framework on the surface of fiber were formed (Fig. S2, 16 h), and some features of the natural Salix precursor were still retained, because the reaction time was limited. With the reaction time further extended, water slowly seeped into the cellulose crystalline region, and a large number of the cellulose chain was damaged and broken. As a result, the original framework structure of non-dissolved cellulose and lignin were almost all disrupted and underwent heterogeneous pyrolysis-like process and formed the unique molecular sieve-type pore structure. This unique pore structure of hydrochar is based on structure of nature Salix.

It is well known that the specific surface area, pore structure and surface functional groups are key factors influencing the adsorption of metal ions. HC-26 contains a large amount of oxygen and nitrogen surface functional groups, which have a high affinity toward to metal ions. Therefore, they constitute the majority of the adsorption sites for Cr (VI). On the other hand, these surface functional groups could be protonated under acidic condition, which had strong electrostatic attraction with anions (HCrO^4−^ or Cr_2_O_7_^2−^). At the same time, the hydorchar possesses a molecular sieve-type open pore structure, which provided a number of active binding sites for the adsorption of Cr (VI)^26^. Thereafter, HC-26 hydrochar was applied as a representative absorbent for studying Cr (VI) adsorption performance in aqueous solution. For the purpose of comparison with HC-26, the removal efficiency of Cr (VI) has been assessed by the commercial activated carbon and corn stalk-based hydrochar, which are prepared under the same condition as HC-26. The results indicated that HC-26 exhibited the best performance for Cr (VI) removal in aqueous solution among these three adsorbents (see supporting information S3) due to its specific surface chemical and physical properties. Thus, a series of experiments have been designed and conducted with the present of HC-26 hydrochar.

### Effect of pH on Cr (VI) Adsorption

Both the surface functional groups present on the adsorbent and the Cr ions’ form in aqueous solution were affected by the pH of the solution. The effect of pH on the removal of Cr (VI) was examined by using Cr (VI) initial concentration of 50 mg L^−1^ and adsorbent dosage of 50 mg, (Fig. 3 (a)). The results showed that the adsorption of Cr (VI) was closely related to the pH values in aqueous solutions. The adsorption efficiency of hydrochar was found to decrease drastically with the increase of pH ranging from 1.0 to 9.0, and the maximum adsorption efficiency (99.84%) was achieved at pH 1. The adsorption efficiency was influenced by electrostatic attraction between functional groups on hydrochar surface and the Cr (VI) anions species in aqueous solutions, while the surface charge of the adsorbents, the ionization degree of chromium species and chromium ionic forms are all depended on pH of the solution. Cr (VI) ion exists in the form of Chromic acid (H_2_CrO_4_), chromate (CrO_4_^2−^), dichromate (Cr_2_O_7_^2−^) and hydrogen chromate (HCrO^4−^) in solution. H_2_CrO_4_ predominates at pH less than about 1.0. In acidic media (pH 1.0–6.0), Cr_2_O_7_^2−^ and HCrO_4_^−^ ions are the dominant species, and under alkaline conditions (pH > 8.0), only CrO_4_^2−^ions are stable in solution. In an acidic environment (pH 1.0–6.0), multiple functional groups such as −COOH, −OH and N-containing functional groups existed on the surface of hydrochar, that is, the surface is surrounded by a large number of H^+^ ions and has a positive electrical load, which attracted the HCrO_4_^−^ and Cr_2_O_7_^2−^. Under alkaline conditions (pH > 8.0), as OH^−^ concentration increases, the hydrochar surface involves negative electrical load, and the excessive OH^−^ on the surface of hydrochar compete with the CrO_4_^2−^ in term of adsorption, and then, resulting in the low adsorption efficiency of hydrochar.

### Effect of Adsorbent Dosages

The influence of adsorbent dosages on the Cr (VI) adsorption was examined by varying dosages from 5 to 120 mg at a pH of 1.0, and the results were shown as Fig. 3(b). As the adsorbent dose increases, the removal efficiency for Cr (VI) rapidly increases in the initial stages but hardly changes in the later stages. For definite number of Cr (VI), this was due to the increased dosage of the adsorbent resulting from the more functional groups, or increased adsorbent surface area and active binding sites^44^. Therefore, when the critical dosage has been reached, the removal percentage remains constant. i.e. the optimum hydrochar dosage for maximum adsorption is 60 mg.

### Effect of Initial Concentration of Chromium

As Fig. 3(c) displayed, the kinetic profiles of Cr (VI) and total Cr concentration were obtained when hydrochar was mixed with Cr (VI) solutions at various initial Cr (VI) concentrations. The effect of initial concentration of chromium ions on the adsorption capacity was investigated in a range from 10 to100 mg L^−1^ at pH 1.0 for a period of 15 h. Hydrochar adsorbed the Cr (VI) completely at initial Cr (VI) concentrations of up to 70 mg L^−1^. Its removal efficiency for Cr (VI) was increased with the increase of initial concentration, which is that more Cr (VI) ions in the solution have been absorbed onto the binding sites on the surface of hydrochar. However, the removal efficiency of Cr (VI) was decreased by further increasing the initial concentration of Cr (VI), because the adsorbent had a limited number of active sites, which were saturated in a certain concentration.

### Adsorption Kinetic

Figure 3(d) presented the effect of contact time on Cr (VI) adsorption by changing the contact time from 20 to 1080 min. The plot showed that an initial rapid phase and a second slower equilibrium phase were found based on the rate of Cr (VI) adsorption. The results indicated that the Cr (VI) could be removed rapidly at the beginning. While the adsorption equilibrium was established when the contact time reached 300 min, and then the value of adsorption keep a constant with further increasing time. Hence 300 min was considered as the equilibrium time. The higher rate of adsorption at initial stages could be attributed to available numbers of free functional groups, and the Cr (VI) could transfer easily to the hydrochar surface and diffuse to the internal adsorption sites through the pores. As the system reached equilibrium, the accumulation of Cr (VI) resulted in limited mass transfer of the Cr (VI) from the liquid phase to the external surface of the adsorbent, and then intraparticle diffusion which became predominant with the time increased^45^.

In order to assess the adsorption rates in the overall adsorption process of Cr (VI) onto hydrochar, the kinetic studies for Cr (VI) adsorption on the hydrochar were carried out by varying the contact time at three different temperatures. The adsorption process was investigated by the kinetic models of pseudo-first order and pseudo-second order (see the equations (1) and (2) in supporting information). The kinetic parameters for the above models are summarized in Table 2 and the kinetic fitting plots are shown in Figs 4,5 respectively. The values of R^2^ for pseudo-second order are close to 1 (greater than 0.99). Higher R^2^ values for the pseudo-second-order model clearly were given for the assayed kinetic models at the temperatures of 20 °C, 30 °C and 40 °C. Moreover, the theoretical q_e_ values are almost consistent with the experimental q_e_ values in pseudo-second order model. Thus, the experimental data are in good agreement with the pseudo-second-order model (see Fig. 5), which implied that the adsorption behavior of hydrochar was similar to chemical adsorption, therefore, the rate-limiting step could be explained by the chemical electrostatic interactions.

**Table 2:** Kinetic parameters of the pseudo-first-order and pseudo-second-order models for the adorption of Cr (VI) on hydrochar.

T (°C)	pseudo-first-order model			pseudo-second-order model		
	q_e_ (mg g^−1^)	k_1_ (min^−1^)	R^2^	q_e_ (mg g^−1^)	k_2_ (g mg^−1^·min^−1^)	R^2^
20	5.60	0.0075	0.9102	25.36	0.0032	0.9998
30	8.76	0.0189	0.9462	25.98	0.0034	0.9998
40	2.38	0.0202	0.8164	25.22	0.0173	0.9999

### Adsorption Isotherms

**Figure 6:**
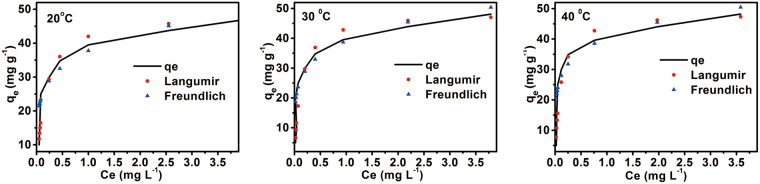
Adsorption isotherms for adsorption of Cr (VI) onto the hydrochar fitted to the Langmuir and Freundlich isotherm models at different temperature.

To further evaluate the adsorption capacity of the Cr (VI) and adsorption mechanism, and describe the relationship between the surface properties, affinity of hyrochar and Cr (VI) Langmuir and Freundlich isothermal models (see the equation (3) and (4) in supporting information)were used to describ a monolayer homogeneous and multilayer heterogeneous adsorption respectively. The Langmuir model has been widely used to estimate the maximum adsorption capacity which was impossible to reach experimentally. The Langmuir equation is obeyed by the adsorption equilibrium, the slope and the intercept in line of C_e_ versus C_e_/q_e_ give the values of q_m_ and b, while the plot of lnq_e_ versus lnc_e_ was employed to generate the intercept value of K_f_ and the slope of 1/n from Freundlich equation. The parameters of two models at 20–40 °C are summarized in Table 3 and the adsorption isotherms are shown in Fig. 6. It could be observed that the relative parameters R^2^ of Langmuir are larger than that of Freundlich at all temperature and the Langmuir model could describe the adsorption equilibrium data more precisely (see Fig. 6). The maximum adsorption capacity of the hydrochar for Cr (VI) was found to be 0.976 g g^−1^ at temperatures 40 °C. These results suggest that the adsorption behaviors mainly belong to monolayer adsorption, and the separation factor R_L_ also can express the essential characteristics of the Langmuir model (see the equation (5) in supporting information). However, the R^2^ values of the Freundlich model were also quite high, implying that multilayer heterogeneous adsorption was involved in adsorption process. The results in Table 3 showed that the values of 1/n are between 0.1 and 0.4, and gradually decrease with the increase of temperature, which indicated that high temperature was more preferable for the Cr (VI) adsorption. Thus, the adsorption was a complex multistep process, due to the complexity of chemical structure and physical property of hydrochar. The different oxygen and nitrogen containing functional groups resulted in numbers of different active sites on the hydrochar surface, where some adsorption likely occurred via the interactions such as electrostatic force, coordination and hydrogen bonding between the Cr (VI) and the adsorbent, and residue Cr (VI) ions may also be adsorbed into the pores of adsorbent through physical adsorption.

**Table 3:** Fitting parameters of the Langmuir and Freundlich equations for the adsorption of Cr (VI) on hydrochar at different temperature.

Langmuir model				Freundlich model		
T (° C)	q_m_ (mg g^−1^)	Kc (L g^−1^)	R^2^	K_f_ (L g^−1^)	n	R^2^
20	48.3	6.41	0.9975	37.781	5.289	0.9330
30	48.6	7.88	0.9962	39.169	5.956	0.9949
40	48.7	9.42	0.9970	40.436	5.764	0.9205

To confirm the changes of internal energy of adsorption process and deduce whether the process is spontaneous (see the equation (6), (7), (8) in supporting information), the ΔG, ΔH, and ΔS were calculated and listed in Table S4. The ΔG values at each temperature were negative and decreased with increase in temperature, demonstrating that higher temperature facilitates the adsorption of Cr (VI) onto hydrochar because the adsorption process is spontaneous. The standard enthalpy and entropy changes of adsorption were calculated to be 69.19 kJ mol^−1^ and 0.31 kJ mol^−1^ respectively. The positive value of ΔH indicated the adsorption process was endothermic, hence the amount of Cr (VI) adsorbed on hydrochar increase with increasing temperature of the solution. The ΔS value was also positive, indicating that the adsorption of Cr (VI) ions on hydrochar increased disorder at solid-liquid interface.

## Conclusion

In summary, hydrochars have been prepared from salix by HTC. The results showed that the hydrochar is a high-efficiency adsorbent for the removal of Cr (VI) from aqueous solution, and a large amount of oxygen and nitrogen surface functional groups have been founded in HC-26, which have a high affinity toward to metal ions. Furthermore, surface functional groups could be easily protonated under acidic conditions, they could attract anions (HCrO^4−^ or Cr_2_O_7_^2−^) by electrostatics. At the same time, the hydorchar possesses a molecular sieve-type open pore structure, which provided a number of active binding sites for the adsorption of Cr (VI). Adsorption of Cr (VI) was found to be effective in the lower pH, and the maximum removal efficiency (99.84%) was achieved at pH of 1 and temperature of 20 °C. The pseudo-second-order kinetics and Langmuir isotherm model can well describe the Cr (VI) adsorption process. This distinguished adsorption performance reflects the great potential applications of Salix-based hydrochar in term of Cr (VI) removal.

## Experimental Section

### Preparation for Hydrochar

Salix psammophila were collected from the desert of western Inner Mongolia (China), cut into small chips, milled and sieved to particles below 100 μm. The other chemicals used in the study were reagent grade.

2 g dried Salix sample was dispersed in water (65 ml) and stirred for 2 h at room temperature. The mixture was then placed in a Teflon-lined autoclave (100 ml), and heated up to 220 °C at a heating rate of around 3 °C/min, and reacted for 26 h. After the reaction, the reactor was cooled down to room temperature, and then the solid/liquid mixture was filtered; the solid fraction (here denoted as hydrochar) was washed repeatedly with distilled water and ethanol, then was dried at 80 °C for 24 h, and the grounded powder was used for various adsorption experiments. The hydrochars obtained from different reaction time were denoted as HC-t, where t represents the reaction time, for instance, HC-6 represents an HC obtained after reaction for 6 h.

### Adsorption Experiments

The effects of operating conditions (pH, adsorption time, adsorbent dosages, initial Cr (VI) concentration, and adsorption temperature) on Cr (VI) removal were studied, where the pH was adjusted by adding either 0.01 N HCl or 0.01 N NaOH solution. The residual concentration of the Cr (VI) was measured by UV–vis spectrophotometer at 540 nm using the diphenylcarbazide method. The sorption kinetic studies were conducted with an initial Cr (VI) solution concentration of 50 mg L^−1^ and 0.5 g hydrochar at different temperature of 20–40 °C for different contact times. Adsorption isotherms were recorded over a concentration range of 10–100 mg L^−1^ of Cr (VI) solution with 0.5 g adsorbent dosage at different reaction temperature of 20–40 °C for 8 h. The adsorption capacity of the Cr (VI) ions (q_e_) was determined by equation (1):

1 $${q}_{e}=({C}_{0}-{C}_{e})\frac{V}{m}$$

where C_0_ and Ce (mg L^−1^) are concentrations of Cr (VI) solution at initial stage and equilibrium conditions, V (L) is volume of the Cr (VI) solution and m (g) is mass of the adsorbent used.

To evaluate the removal efficiency, the percentage of removal of chromium ions was calculated as equation (2):

2 $${\rm{Removal}}\,( \% )=\frac{{C}_{0}-{C}_{e}}{{C}_{0}}\times 100$$

## Electronic supplementary material

supplementary information

## References

[1] Zou Y (2016). Environmental Remediation and Application of Nanoscale Zero-Valent Iron and Its Composites for the Removal of Heavy Metal Ions: A Review. Environ. Sci. Technol..

[2] Aragay G, Pons J, Merkoҫi A (2011). Recent Trends in Macro-, Micro-, and Nanomaterial-Based Tools and Strategies for Heavy-Metal Detection. Chem. Rev..

[3] Bailey SE, Olin TJ, Bricka RM, Adrian DD (1999). A review of potentially low cost sorbents for heavy metal. Water Research..

[4] Ghorbani-Khosrowshahi S, Behnajady MA (2016). Chromium (VI) adsorption from aqueous solution by prepared biochar from Onopordom Heteracanthom. Int. J. Environ. Sci. Technol..

[5] Goswami M, Borah L, Mahanta D, Phukan P (2014). Equilibrium modeling, kinetic and thermodynamic studies on the adsorption of Cr (VI) using activated carbon derived from matured tea leaves. J. Porous Mat..

[6] Owlad M, Aroua MK, Daud WAW, Baroutian S (2009). Removal of Hexavalent Chromium-Contaminated Water and Wastewater: A Review. Water Air Soil. Poll..

[7] Mahapatra A, Mishra BG, Hota G (2013). Studies on electrospun alumina nanofibers for the removal of chromium (VI) and fluoride toxic ions from an aqueous system. Ind. Eng. Chem. Res..

[8] Weng CH (1996). Chemical Interactions between Cr (VI) and Hydrous Concrete Particles. Environ. Sci. Technol.

[9] Gang Q, Michael JM, Blute NK, Seidel C, Fong L (2005). Hexavalent Chromium Removal by Reduction with Ferrous Sulfate, Coagulation, and Filtration:  A Pilot-Scale Study. Environ. Sci. Technol..

[10] Chen XM, Wang DH (2007). Electrically Regenerated Ion Exchange for Removal and Recovery of Cr (VI) from Wastewater. Environ. Sci. Technol..

[11] Bhowal A, Bhattacharyya G, Inturu B, Datta S (2012). Continuous removal of hexavalent chromium by emulsion liquid membrane in a modified spray column. Sep. Purif. Technol..

[12] Martínez-Delgadillo SA, Mendoza-Escamilla VX, Mollinedo-Ponce HR, Puebla H, Méndez-Contreras JM (2011). Effect of the Ultrasonic Irradiation on the Cr (VI) Electroreduction Process in a Tubular Electrochemical Flow Reactor. Ind. Eng. Chem. Res..

[13] Ghaffari A, Husain SW, Tehrani MS, Anbia M, Azar PA (2015). Highly efficient adsorption of hexavalent chromium from the aqueous system using nanoporous carbon modified with tetraethylenepentamine. Int. J. Environ. Sci. Technol..

[14] Xu CH, Zhu LJ, Wang XH, Lin S, Chen YM (2014). Fast and Highly Efficient Removal of Chromate from Aqueous Solution Using Nanoscale Zero-Valent Iron/Activated Carbon(NZVI/AC). Water Air Soil. Poll.

[15] Park D, Yun YS, Park JM (2006). Mechanisms of the removal of hexavalent chromium by biomaterials or biomaterial-based activated carbons. J. Hazard. Mater..

[16] Gao Y, Chen CL, Tan X, Xu H, Zhu KR (2016). Polyaniline-modified 3D-flower-like molybdenum disulfide composite for efficient adsorption/photocatalytic reduction of Cr(VI). J. Colloid Interf. Sci..

[17] Zeng YB, Woo H, Lee G, Park J (2010). Adsorption of Cr (VI) on hexadecylpyridinium bromide (HDPB) modified natural zeolites. Micropor. Mesopor. Mat..

[18] Jia ZG, Wang QZ, Ren DDP, Zhu RS (2013). Fabrication of one-dimensional mesoporous α-Fe_2_O_3_ nanostructure via self-sacrificial template and its enhanced Cr (VI) adsorption capacity. Appl. Surf. Sci..

[19] Liu CC (2006). Chromium Removal and Sorption Mechanism from Aqueous Solutions by Wine Processing Waste Sludge. Ind. Eng. Chem. Res..

[20] Huang XX (2016). Effective removal of Cr (VI) using b-cyclodextrin–chitosan modified biochars with adsorption/reduction bifuctional roles. RSC Adv..

[21] Deng SB, Ting YP (2005). Polyethylenimine-Modified Fungal Biomass as a High-Capacity Biosorbent for Cr (VI) Anions:  Sorption Capacity and Uptake Mechanisms. Environ. Sci. Technol..

[22] Mallampati R, Li X, Valiyaveettil S, Valiyaveettil S (2015). Fruit Peels as Efficient Renewable Adsorbents for Removal of Dissolved Heavy Metals and Dyes from Water. ACS Sustainable Chem. Eng..

[23] Daoud W, Ebadi T, Fahimifar A (2015). Optimization of hexavalent chromium removal from aqueous solution using acid-modified granular activated carbon as adsorbent through response surface methodology. Korean J. Chem. Eng..

[24] Yu TL (2016). Synthesis of novel aminated cellulose microsphere adsorbent for efficient Cr (VI) removal. Radiat. Phys. Chem..

[25] Zhu Y, Zhang H, Zeng H, Liang M, Lu R (2012). Adsorption of chromium (VI) from aqueous solution by the iron (III)-impregnated sorbent prepared from sugarcane bagasse. Int. J. Environ. Sci. Technol..

[26] Kera NH (2016). Selective removal of Cr (VI) from aqueous solution by polypyrrole/2,5-diaminobenzene sulfonic acid composit. J. Colloid Interf. Sci..

[27] Hu B, Yu SH, Wang K, Liu L, Xu XW (2008). Functional carbonaceous materials from hydrothermal carbonization of biomass: an effective chemical process. Dalton Trans..

[28] Libra JA (2011). Hydrothermal carbonization of biomass residuals: a comparative review of the chemistry, processes and applications of wet and dry pyrolysis. Biofuels..

[29] Titirici MM, White RJ, Falcoa C, Sevill M (2012). Black perspectives for a green future: hydrothermal carbons for environment protection and energy storage. Energy Environ. Sci..

[30] Liu SX, Sun J, Huang ZH (2010). Carbon spheres/activated carbon composite materials with high Cr (VI) adsorption capacity prepared by a hydrothermal method. J. Hazard. Mater..

[31] Liu ZG, Zhang FS, Wu JZ (2010). Characterization and application of chars produced from pinewood pyrolysis and hydrothermal treatment. Fuel..

[32] Han M (2017). from corncob prepared via hydrothermal carbonization and post-pyrolysis method. *Sci*. Rep-UK..

[33] Alatalo SM (2015). Versatile Cellulose-Based Carbon Aerogel for the Removal of Both Cationic and Anionic Metal Contaminants fromWater. ACS Appl. Mater. Inter..

[34] Liu YH (2013). Removal of uranium from aqueous solution by a low cost and high-efficient adsorbent. Appl. Surf. Sci..

[35] Łukaszewicz JP, Wesołowski RP (2008). Fabrication of molecular-sieve-type carbons from Salix viminalis. Micropor. Mesopor. Mat..

[36] Reichardt P, Merken H, Clause TP (1992). Phenolic glycosides from Salic Lasiandra. J. Nat. Prod..

[37] Lei YQ, Su HQ, Tian RK (2016). Morphology evolution, formation mechanism and adsorption properties of hydrochars prepared by hydrothermal carbonization of corn stalk. RSC Adv..

[38] Zhu, X. D. *et al*. A novel porous carbon derived from hydrothermal carbon for efficient adsorption of tetracycline. *Carbon*. 627-636 (2014).

[39] Calucci L, Rasse DP, Forte C (2013). Solid-State Nuclear Magnetic Resonance Characterization of Chars Obtained from Hydrothermal Carbonization of Corncob and Miscanthus. Energ. Fuel..

[40] Baccile N (2009). Structural Characterization of Hydrothermal Carbon Spheres by Advanced Solid-State MAS ^13^C NMR Investigations. J. Phys. Chem. C..

[41] Wohlgemuth SA, Vilela F, Titirici MM, Antonietti M (2012). A one-pot hydrothermal synthesis of tunable dual heteroatom-doped carbon Microspheres. Green Chem..

[42] Sevilla M, Fuertes AB (2009). Chemical and structural properties of carbonaceous products obtained by hydrothermal carbonization of saccharides. Chemistry (Weinheim an der Bergstrasse, Germany).

[43] Biswas AK, Umeki K, Yang W, Blasiak W (2011). Change of pyrolysis characteristics and structure of woody biomass due to steam explosion pretreatment. Fuel Process. Technol..

[44] Jiang ZJ (2015). Adsorption of hexavalent chromium by polyacrylonitrile (PAN)-based activated carbon fibers from aqueous solution. RSC Adv..

[45] Tirtom VN, Dincer A, Becerik S, Aydemir T, Celik A (2012). Removal of lead (II) ions from aqueous solution by using cross linked chitosan clay beads. Desalin. Water Treat..

