# Supplementary material for: A Novel Nitrogen Enriched Hydrochar Adsorbents Derived from Salix Biomass for Cr (VI) Adsorption
Source: Sci Rep. 2018 Mar 6;8:4040. doi: 10.1038/s41598-018-21238-8 (PMC5840138; doi:10.1038/s41598-018-21238-8)
Supplement: Supplementary file 1 — supplementary information [file 41598_2018_21238_MOESM1_ESM.pdf]

A Novel Nitrogen Enriched Hydrochar Adsorbents Derived from Salix Biomass for Cr (VI) Adsorption

Yanqiu Lei, Haiquan Su*, Fuli Tian
School of Chemistry & Chemical Engineering, Inner Mongolia University, 235 West College Road, Hohhot, 010021, Inner Mongolia, China.
* Corresponding author: School of Chemistry & Chemical Engineering, Inner Mongolia University, 235 West College Road, Hohhot, 010021, Inner Mongolia, China. E-mail: haiquansu@yahoo.com; Fax: 86-471-4992979; Tel: +86-471-4992979

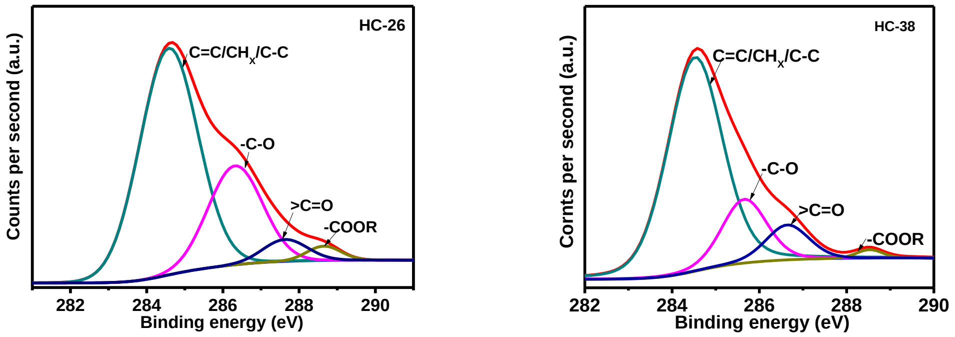

S1. C1s core-level of the hydrochars for 26 h and 38 h.

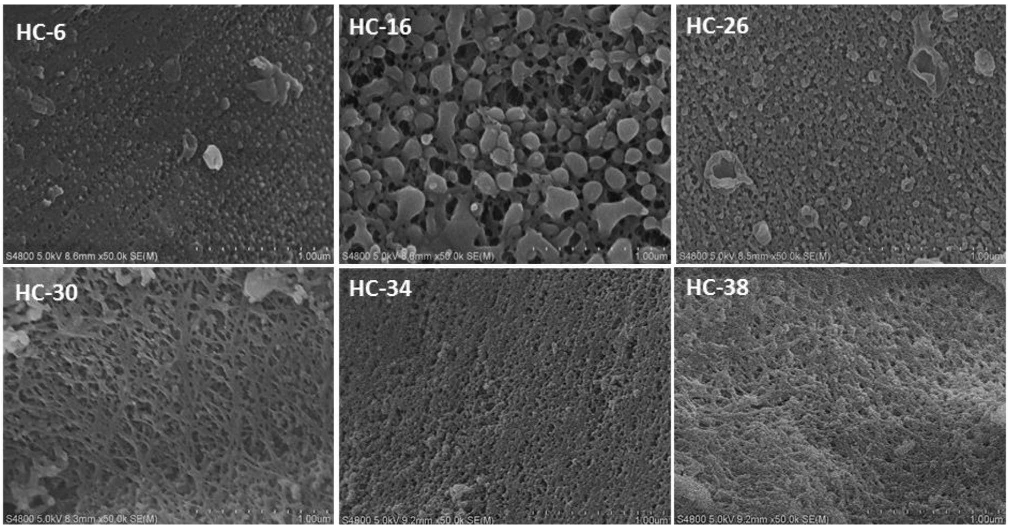

S2. SEM microphotographs of the hydrochars obtained at different time.

Adsorption Kinetic

The adsorption process was investigated by the well-known kinetic models of pseudo-first order and pseudo-second order. Which are expressed as follows:

$$\ln(q_e - q_t) = \ln q_e - k_1 t \qquad (1)$$

$$\frac{t}{q_t} = \frac{t}{q_e} + \frac{1}{k_2 q_e^2} \qquad (2)$$

Where q_t and q_e (mg g^{-1}) are the amount of Cr (VI) at time t and equilibrium, k_1 (min^{-1}), k_2 (g mg^{-1} min^{-1}) are the rate constants of pseudo-first order and pseudo-second order, respectively.

Adsorption Isotherms

The equations Langmuir and Freundlich isothermal models are given as follows:

The Langmuir model:

$$\frac{C_e}{q_e} = \frac{C_e}{q_m} + \frac{1}{q_m K_C} \qquad (3)$$

The Freundlich model:

$$\ln q_e = \ln k_f + \frac{1}{n}\ln C_e \qquad (4)$$

Where C_e (mg L^{-1}) and q_e (mg g^{-1}) are the Cr (VI) concentration at equilibrium and the absorbed amount of Cr (VI) on hydrochar at equilibrium, respectively, q_m (mg g^{-1}) is the maximum adsorption capacity, K_C (L g^{-1}) and K_f (L g^{-1}) are the Langmuir and Freundlich constants reflecting adsorption capacity and adsorption strength, and 1/n is relative to the adsorption intensity.

The separation factor R_L can express the essential characteristics of the Langmuir model, R_L is calculated using the following equation:

$$R_L = \frac{1}{1 + C_0 b} \qquad (5)$$

Where C_0 (mg L^{-1}) is the initial concentration of the adsorbate in solution, b (L mg^{-1}) is the Langmuir isotherm coefficients. The value of R_L indicates the adsorption isotherm process is unfavorable (R_L> 1), linear (R_L=1), favorable (0 < R_L< 1), irreversible (R_L=0). The obtained R_L values fall in the range of 0-1, indicating that the adsorption process was favorable.

thermodynamic parameters:

To confirm the changes of internal energy of adsorption process and deduce whether the process is spontaneous, thermodynamic parameters were calculated using the following equation:

$$k_C = \frac{q_e}{C_e} \qquad (6)$$

$$\Delta G = -RT\ln K_c \qquad (7)$$

$$\ln K_c = \frac{\Delta S}{R} - \frac{\Delta H}{RT} \qquad (8)$$

Where K_C (L mol^{-1}) is the Langmuir constant. T (K) is the absolute temperature and R (8.314 J mol^{-1} K^{-1}) is the universal gas constant. ΔH and ΔS was calculated from the slope and intercept of a plot of $\ln K_C$ versus 1/T.

S3.The adsorbing capacity of different type carbon adsorbent on the adsorption of Cr (VI).

Adsorbent type	removal %	SBET (m^2 g^{-1})
HC-26	99.84	15.1
Corn stalks -hydrochar	67.26	12.0
Actived carbon	31.22	801.5

S4. Thermodynamic parameters (ΔG, ΔH, ΔS) for adsorption of Cr (VI) on hydrochar at different temperature.

T(^{o}C)	K_C (L mol^{-1})	ΔG (kJ mol^{-1})	ΔS (J mol^{-1} K^{-1})	ΔH (kJ mol^{-1})
20	9.03	-4.35	0.31	69.19
30	9.77	-4.44		
40	10.33	-5.74		
45	11.24	-6.38		

The results indicated that HC-26 exhibited the best performance for Cr (VI) removal in aqueous solution among these three adsorbents.
